# A History of Maryland's New Dental Law, 1896

**Published:** 1897-02

**Authors:** Richard Grady

**Affiliations:** Baltimore.


ARTICLE VII.

### A HISTORY OF MARYLAND'S NEW DENTAL LAW, 1896.

RICHARD GRADY, M. D., D. D. S., BALTIMORE.

It had been my intention (until requested by Dr. Noble, chairman, to confine my paper to the history of the new Maryland dental law) to consider remarks in private, and current printed expressions to the effect that the State is taking altogether too parental an attitude towards its citizens, in assuming to act as the guardian of the poor and ignorant, and in preventing men from receiving due benefit from their business ability. What the State has done in the field of public health need only be mentioned. Druggists, dentists, physicians and plumbers, in Maryland, are all under the regulation of law. If persons do not know enough to protect themselves against incompetent practitioners in dentistry, it is argued, the ignorant ought to suffer. But we, as dentists, are sufficiently far advanced to understand plainly that the comprehension of government as simply a restraining legal hand at the throat of would-be-criminals is far too small to meet the situation. We can see that it covers many acts for the common good, not the least of which is the exercise of the police power for the protection of the people and, incidentally, the benefit of the dental profession.

### A REVIEW.

Any history of the dental law of 1896 should follow a review of the influences that affected its preparation.

The history of dental legislation in Maryland goes back to 1879. In September of that year began the movement for regulating the practice of dentistry. The sentiment then expressed was, "Require of the practitioner first a diploma, and then an examination; a diploma does not show that its holder is qualified to meet whatever demands may be made upon him in his profession."

In 1882 a bill was introduced, which was printed and passed to a second reading in the House of Delegates, but as it was only reported from the Judiciary Committee (March 25, 1882) about one week before the close of the session it did not reach the Senate.

In January, 1884 two bills were presented to the Legislature of Maryland, by opposing organizations. Both bills had in view the desire to awaken the public to the fact that they are daily preyed upon by persons who make pretensions to having what they do not possess, and were not framed for the pecuniary benefit of dentists as a class. One provided that "the board of examiners shall consist of five dental graduates appointed by the Governor from ten candidates, whose names shall be handed him by the Dental Legislative Society of Maryland." Neither bill was adopted in its entirety.

Nevertheless a dental law was passed and a board of dental examiners created "to consist of five reputable practicing dentists appointed by the Governor, from the dentists residing in the State of Maryland." This act, approved March 31, 1884, made punishable only "willful" violations of the law. It called forth criticisms from the Pennsylvania State Dental Examining Board, the National Association of Dental Examiners, and several dental journals, because of a restrictive provision that "it shall be unlawful for any one to engage in the practice of dentistry unless he shall first have passed a satisfactory examination before the Board of Examiners or shall hold a diploma from a university or college chartered by or under the laws of Maryland." Exception was made in the act to persons who were engaged in the practice of dentistry in Maryland before its passage.

Acting upon these protests, the Dental Examiners of Maryland submitted amendments, approved by the dentists of the State, which placed the Maryland dental law in harmony with the dental laws of other States, and these the Legislature of 1886 passed without the change of a single word.

In 1888 the authorized code of public general laws superseded all the statute laws of the State of Maryland and the dental law was again changed in these particulars:

(1) The law had no application to persons engaged in the practice of dentistry prior to March 31, 1884.

(2) As to all other persons it was unlawful for them to practice without a certificate or diploma.

(3) It was uncertain as to cases occurring prior to the adoption of the Code whether certificates could be gotten by mere registration, or by examination before the board.

(4) One who was practicing without a certificate must be shown to have wilfully violated the law.

In 1890, it having been shown that the dental law was not exyressed in precise, accurate terms about which there could be no dispute, a bill was drafted and presented to the Legislature, but failed to reach a vote. This bill was presented from a mass-meeting of the dentists of the State.

In 1892, another bill was introduced. The editorial press criticised and opposed the bill as "inartificially constructed and badly put together," saying, "This bill is monopoly pure and simple"--"it is in fact opening up a new field for trusts,"—"the dental bill reaches its insidious fingers back eight years into the past and gives a dental board formed by a fearful and wonderful method the power to close up the office of any dentist who has gone into the profession within the past eight years"—"now when our colleges are provided with learned and experienced faculties and all the facilities which modern science commands, it is both superfluous and absurd to set up state boards of examination to review their work. Under the best conditions such a review could not be fairly done. In fact the boards themselves are very rarely composed of competent men, no matter whether chosen by associations, or by political discretion. The best class of practitioners will have nothing to do with them."

In 1894, a bill passed the Legislature of Maryland (chiefly through the individual efforts of Dr. Wm. Shaw Twilley), but it was vetoed by the Governor.

No greater tribute can be paid to the framers of the law of 1884 (as amended in 1886 and codified in 1888) than to say that in the twelve years of its administration, the act never required construction by any court, and never met with decided opposition.

### THE DENTAL LAW OF 1896.

About a year ago, in the Maryland State Dental Association, on Dr. B. Holly Smith's motion, a committee was appointed "to draft an acceptable dental law and have it passed." At the meeting of the committee Dr. T. S. Waters was elected chairman and a sub-committee of three was entrusted with the preparation of a bill. Weekly meetings were held by the sub-committee, continuing through months. Finally before the close of 1895 a report was made to the general committee. Believing that the proposed bill could be further improved it was recommitted to the sub-committee, who reported it substantially as it passed the Legislature, excepting discretion in examining applicants, and legal phraseology.

### A COMPARISON.

*Scope of the Act of 1896 compared with the law of 1884, as will appear from the following analysis.*

1. The law of 1884 had no application to persons engaged in the practice of dentistry at the time of the passage of the Act: the law of 1896 excepts only dentists holding certificates duly issued to them prior to April 4, 1896, and does not prevent the punishment of persons who may have offended against any of the provisions of the law of 1884, as amended in 1886, and codified in 1888.

2. The law of 1896 forbids a minor, or an unlicensed person, to practice dentistry except as a dental student, operating under the immediate supervision of his instructor in a dental infirmary of a dental school.

3. The law of 1884 permitted any and all persons to appear before the Dental Board for examination; and excepted holders of diplomas from Maryland Dental Schools from examination: the law of 1896 prescribes special qualifications for a licentiate (a) he must be 21 years of age; (b) he must be a regular graduate in dentistry; (c) he may be examined in the discretion of the Board.

4. No greater particularity in the indorsement of diplomas is noted in the law of 1896 than in the amendment of 1886, to prevent careless registration.

5. The law of 1896 defines the practice of dentistry.

6. The law of 1884 required that one who was practicing without a certificate must be shown to have wilfully violated the law: the law of 1896 omits "wilfully," and includes confinement in Baltimore City Jail.

7. Article 32 of the Code of Public General Laws is repealed specifically by title thus avoiding any question that might arise as to repeal by implication.

8. The anomaly of subjecting dental graduates to an examination by those who had not passed through a curriculum, and the doubtfulness of the word "reputable" in the law of 1884, together with the desire to make the selection of the Dental Board a professional rather than a political matter and to keep a majority of experienced members on the Board at all times, led to three changes in the law of 1896: (a) a board of "six practicing dentists of recognized ability and honor;" (b) "who have held regular diplomas for five years;" (c) "chosen by a majority vote of the State Dental Association" but "appointed by the Governor out of a list of nine names; (d) one-third of the board retiring biennially as does the Senate of the United States; (e)

suspending any member absent from two successive regular meetings, as in the past four years, one member had been present but once, and another member twice; and for lack of a quorum no annual reports were made in 1891 and 1892. It was no longer necessary to incorporate into the law of 1896 as in that of 1884 that the Governor "shall select them from the dentists residing in the State" (Maryland) because at that time (1884) it was publicly charged that the only opposition to the bill presented by the Maryland State Dental Association came from "the members of a dental society styled the Maryland and District of Columbia Dental Association, the majority of the active members of which are citizens and residents of the District of Columbia, and who are not legally entitled to carry out the provisions of any Act regulating the practice of dentistry in the State of Maryland."

9. By the law of 1884 one member could grant any certificate to any applicant, and the charge for either a permanent or temporary certificate was one dollar; by the law of 1896 (a) the officers of the board issue temporary certificates; (b) the applicants holding regular dental diplomas (c) duly registered by *a* board of dental examiners; (d) fee, five dollars.

10. By the law of 1884, if no certificate was issued after examination (and the examination might occupy a week) no fee was collected; by the law of 1896 "a fee of ten dollars shall be paid to the secretary of the board by any applicant for examination and registration, which money shall be used towards paying the expenses of the board."

11. By the law of 1884 certificates and diplomas granted according to the law were *prima facie* evidence of the right of the holder to practice dentistry in the State of Maryland; by the law of 1896 the certificates must have been duly issued prior to April 4, 1896.

### OBJECTIONS.

Having shown the scope of the measure (which has been much denounced, much defended, but very little studied) it only remains to cite the points brought against the bill (1) before the Maryland State Dental Association; (2) the Committee on Hygiene of the Legislature; (3) the Governor of Maryland, These are briefly:

(a) *The bill permitted dentists pecuniarily connected with dental schools to become members of the dental board.* This objection was overcome by the adoption of a standing resolution by the State Dental Association, making members of dental faculties ineligible for choice by the Association. It was feared that the incorporation of such an amendment into the dental bill, which was on its third reading in the House of Delegates, might jeopardize its passage.

(b) *The discretion left the Board about examinations, and the fear that it might discriminate against a particular school of dentistry.* It by no means follows that because a man possesses a diploma he is qualified to practice dentistry, and the public is entitled to demand that no one be permitted to practice until he is able to stand the test of an examination by men known in the community for their character and professional standing. The public has nothing to do with rivalries and jealousies, but it may properly insist that no man shall be permitted to practice merely because he is able to exhibit a dental diploma which may or may not certify truthfully to his requirements. That the board would undertake to exclude from the profession graduates who prove themselves competent, because they happen to graduate from a certain school, is not to be seriously entertained. When, however, it is suspected that the possession of a diploma is not the guarantee of professional attainments, a rigid examination is the only safeguard for the public.

(c) *Making it unlawful for dental colleges or infirmaries by its professors, demonstrators, assistants or students to receive pay for services rendered patients in the institutions from April to October, that is during the summer session.*

(d) *Making a diploma a prerequisite for examination.* The answer to this was that, in the past twelve (12) years the law had been very liberal, as any person who had desired to practice dentistry in Maryland, without first securing a diploma, could have been examined as to his knowledge and skill in dental surgery; that the object of the present bill was principally for the welfare and protection of the people in the future; that there ought to be no shorter road to enter the profession than the colleges offered.

THE NEW BOARD.

It is harder to use success, than to win it. Twelve years ago the Maryland State Dental Association endeavored to have the appointment of the State Board of Dental examiners; it has now practically succeeded. The Board will do no more than what every educated and honorable dentist in the Association approves; because the members will act under a feeling of direct responsibility to the Association. The men of "ability and honor" have been commissioned. They cannot be too well equipped for their places. Their capacity will be tested. A right conception of their duties will remove criticism. The law was made to protect society against the charlatan and the ignorance of the quack. Most of the friction to be encountered in the execution of the law will arise from over-zeal; on acting without tact or discretion. Where there may have been *intentional apathy*, there may now be *pernicious activity*. It has seemed a piece of good fortune that two members of the board have had experience in executing the law of 1884, and two in forming the law of 1896. After all the great test of what a man can do is what he has done. Previous boards were always a unit on all important questions of policy and principle. It is hoped in every case of violation of the law the board will make a resolute fight. The fact of making the fight, and the further fact that the board is ready to repeat it on provocation will put a stop to the repetition of the offense. The law ought to be carried out in a positive and decisive way and the task of its enforcement ought not to be given up as impossible. It is hoped the members of the board will work in the interests of those who have chosen them and we bid them God speed.—"Read at the Union Meeting of the Maryland State Dental Association and the Washington City Dental Society, 1896."

---

ARTICLE VIII.

### THE BICYCLE AND THE PERINEUM.

